# Human Norovirus Replication in Temperature-Optimized MDCK Cells by Forkhead Box O1 Inhibition

**DOI:** 10.4014/jmb.2003.03071

**Published:** 2020-06-08

**Authors:** Eun-Hye Jeong, Se-Young Cho, Bipin Vaidya, Sang Hoon Ha, Sangmi Jun, Hyun-Joo Ro, Yujeong Lee, Juhye Lee, Joseph Kwon, Duwoon Kim

**Affiliations:** 1 Department of Food Science and Technology and Foodborne Virus Research Center, Chonnam National University, Gwangju 61186, Republic of Korea; 2 Division of Biotechnology, Chonbuk National University, Iksan 54596, Republic of Korea; 3 Biological Disaster Analysis Group, Korea Basic Science Institute, Daejeon 34133, Republic of Korea; 4 Korea Basic Science Institute, Cheongju 28119, Republic of Korea; 5 Convergent Research Center for Emerging Virus Infection, Korea Research Institute of Chemical Technology, Daejeon 34114, Republic of Korea

**Keywords:** Autophagy, forkhead box O1, human norovirus, virus replication

## Abstract

Second, the expression levels of autophagy-, immune-, and apoptosis-related genes at 30°C and 37°C were compared to explore factors affecting HuNoV replication. HuNoV cultured at 37°C showed significantly increased autophagy-related genes (*ATG5* and *ATG7*) and immune-related genes (*IFNA*, *IFNB*, *ISG15,* and *NFKB*) compared to mock. However, the virus cultured at 30°C showed significantly decreased expression of autophagy-related genes (*ATG5* and *ATG7*), but not significantly different major immune-related genes (*IFNA*, *ISG15,* and *NFKB*) compared to mock. Importantly, expression of the transcription factor *FOXO1*, which controls autophagy- and immune-related gene expression, was significantly lower at 30°C. Moreover, *FOXO1* inhibition in temperature-optimized MDCK cells enhanced HuNoV replication, highlighting *FOXO1* inhibition as an approach for successful virus replication. In the temperature-optimized cells, various HuNoV genotypes were successfully replicated, with GI.8 showing the highest replication levels followed by GII.1, GII.3, and GII.4. Furthermore, ultrastructural analysis of the infected cells revealed functional HuNoV replication at low temperature, with increased cellular apoptosis and decreased autophagic vacuoles. In conclusion, temperature-optimized MDCK cells can be used as a convenient culture model for HuNoV replication by inhibiting *FOXO1* and providing adaptability to different genotypes.

## Introduction

Norovirus, a non-enveloped single-stranded positive-sense RNA virus belonging to the *Caliciviridae* family, is classified into seven genogroups (GI–GVII) based on the capsid viral protein (VP)1 sequence [1]. Among them, GI, GII, and GIV cause acute gastroenteritis to humans, and therefore, are collectively referred to as human norovirus (HuNoV), which accounts for more than 95% of all viral gastroenteritis outbreaks worldwide [2, 3]. GI only infects humans; however, GII can infect porcupines and swine, and GIV infects felines and canines as well as humans, thus GII and GIV are considered to have zoonotic potential [1].

The study of HuNoV transmission and the development of effective antiviral interventions are mainly hindered by a lack of an appropriate in vitro culture system. Many cell lines derived from humans and animals cannot support HuNoV replication [4, 5]; for example, viral replication failed in three-dimensional models using human intestinal cells (Int-407 and Caco-2) [6, 7]. However, HuNoV genotypes GI.1, GII.4 and GII.17 were found to replicate in stem cell-derived human intestinal enteroids (HIE) [8], and a strain of GII.4 was reported to replicate in human B cells with enhanced replication by co-incubation with histo-blood group antigen (HBGA)-expressing bacteria [9, 10]. Depending on the genotypes, HuNoV interacts with specific attachment factors such as HBGA or sialoglycan [9, 11]. Although the presence of attachment factor facilitates interaction with the cell surface, the cells may not be susceptible to virus replication. Furthermore, Madin-Darby canine kidney (MDCK) cells were reported to have the well-polarized experimental epithelial cell system for the study of virus infection, and be susceptible to many pathogenic viruses, including canine adenovirus, influenza A, and influenza B virus [12-15]. In addition, even though GIV.2 and GVI are classified as canine norovirus, GI and GII were also reported to be found in the feces of a canine species, such as dog [16], indicating that HuNoV can enter into canine cell lines.

Temperature affects viral replication in host cells by modulating either the rate of synthesis of viral proteins and genomes or the host cell immune response [17-19]. Temperature sensitivity of the viral polymerase activity is usually reported to be responsible for modulating both transcription and replication of virus genome [18]. Apart from this, temperature also regulates host immune response against the virus, which consequently affects viral replication [20]. Furthermore, the transcription factor forkhead box O1 (*FOXO1*) also plays a key role in regulating immune response, and is further associated with the replication of different viruses, including hepatitis B, vesicular stomatitis, and Newcastle disease viruses [21, 22]. However, the effect of temperature and *FOXO1* expression in HuNoV replication has not been reported yet.

Hence, the present study was designed to investigate whether temperature optimization would facilitate HuNoV replication in MDCK cells. Our study demonstrated that MDCK cells can be used as an in vitro model to cultivate HuNoV by modulating temperature via *FOXO1* signaling.

## Materials and Methods

### Cell Lines and Reagents

MDCK cells were purchased from the Korean Cell Line Bank (Korea; KCBL10034). The cells were cultured in Dulbecco’s modified Eagle’s medium (DMEM, Welgene) containing 10% fetal bovine serum (Gibco) and 1% penicillin-streptomycin (100 U/ml; Sigma-Aldrich, USA) at 37°C and 5% CO_2_. For viral infection, the cells were cultured in DMEM supplemented with 100 μg/ml streptomycin, 100 U/ml penicillin, and trypsin treated with 2 μg/ml N*p*-tosyl-L-phenylalanine chloromethyl ketone (TPCK; Sigma-Aldrich). Stock solutions of AS1842856 (Calbiochem, USA) were prepared in dimethyl sulfoxide (Sigma-Aldrich) and stored at −20°C.

### Ethics Statement

Four HuNoV genotypes (GI.8, GII.1, GII.3, and GII.4), isolated separately from HuNoV-positive fecal samples, were obtained from the Gwangju Institute of Health and Environment. All patient information related to the specimens was anonymized for research purposes. The experimental protocol adopted in this study was approved by the Bioethics Review Committee of Chonnam National University (IRB No. 1040198-170420-HR-038-05).

### Virus Stock and Infection

HuNoV specimens isolated from fecal samples were diluted to 10% in PBS (pH 7.4) and stored at −80°C until use. For the infection of MDCK cells, the HuNoV specimens were diluted in infection media consisting of DMEM containing 1% penicillin/streptomycin and 2 μg/ml TPCK. All experiments, excluding a test for adoptability of the culture system to cultivate different HuNoV genotypes, were performed with GII.4 genotype of HuNoV in 48-well plates. The cells were cultured in a 48-well plate at 6.25 × 10^4^ cells/ml at 37°C for 24 h. The cells were infected with 1.91 × 10^2^ genome copies of HuNoV GII.4 for one h, and the cells were then washed with PBS to remove unbound virus. After complete removal, the infected cells were incubated for 72 h at 27°C, 30°C, 34°C, and 37°C in an atmosphere of 5% CO_2_. The infected cells were scraped to extract RNA for virus quantification. To observe the passage of the virus, MDCK cells were cultured in concentrations of 6.25 × 10^4^ cells/ml in 0.75-cm^2^ dishes (48-well plate), 1.375 × 10^5^ cells/ml in 21.50-cm^2^ dishes (60-mm culture dish) and 5.156 × 10^5^ cells/ml in 75-cm^2^ dishes (75T-Flask) for three consecutive passages. The cells were infected with HuNoV by incubation for 72 h in the 1^st^ and 2^nd^ passages and for two weeks in the 3^rd^ passage. For every subsequent passaging, the whole supernatant from the previous passage was used as an inoculum source. To test the adaptability of the culture system for the replication of different genotypes, the cells were infected with 2.28 × 10^2^ virus genome copies of four different genotypes of HuNoV, including GI.8, GII.1, GII.3, and GII.4, and incubated at 30°C for 72 h.

### Quantification of HuNoV VP1 by RT-PCR

To detect HuNoV genome copies in the culture medium by quantitative RT-PCR, 300 μl of the supernatant was collected 72 h after infection. Total RNA was extracted from the supernatant and cells using 700 μl of RNAiso Plus (Takara), according to the manufacturer’s protocol. For cDNA synthesis, 10 μl of RNA and 1 μl of 6-mer random primer (TaKaRa, Japan) were mixed and incubated at 65°C for 10 min; 4 μl of 5 × Moloney murine leukemia virus (M-MLV) reverse transcriptase reaction buffer, 2 μl of 5 × dithiothreitol, 2 μl of dNTP, 0.5 μl of RNase inhibitor (Takara, Japan), and 1 μl of M-MLV were then added, followed by incubation at 37°C for 1 h and 95°C for 5 min using a PCR Thermal Cycler Dice Gradient system. The cDNA was prepared using the HuNoV *VP1* gene primer pair JJV2F (5′-CAA GAG TCA ATG TTT AGG TGG ATG AG-3′) and COG2R (5′-TCG ACG CCA TCT TCA TTC AC-3′) and probe primer RING2P (5′-FAM-TGG GAG GGC GAT CGC AAT CT-Black Hole Quencher) [23]. Quantitative analysis of the number of viral particles was carried out with Thermal Cycler Dice Real Time System III, and the titer was calculated by substituting the cycle threshold value from the qRT-PCR result as the x value in the equation y = −3.498x + 42.305; R^2^ = 0.9974 was calculated from the HuNoV standard curve. The fold change of HuNoV copy number was calculated relative to the initial inoculum of the virus. The viral genome in the initial inoculum was detected after 1 h incubation and washing with PBS.

### Transmission Electron Microscopy

Norovirus-infected cells were detached from the plates using a cell scraper, then collected by centrifugation, and the cell pellets were fixed in 2.5% glutaraldehyde (Electron Microscopy Sciences, USA) in 0.1 M phosphate buffer (pH 7.2) for 3 h at 4°C. The pellet was then washed in several changes of 0.1 M phosphate buffer to remove excess glutaraldehyde. Fixed samples were post-fixed in 1% osmium tetroxide (Electron Microscopy Sciences) in 0.1 M phosphate buffer for 1–2 h in the dark at 4°C. After osmium fixation, the pellets were washed 2–3 times with 0.1 M phosphate buffer and dehydrated through a graded series of ethanol (50%, 70%, 75%, 90%, 95%, and 100%). Dehydrated samples were exposed to two changes of propylene oxide, and then immersed in 2:1 (1 h), 1:1 (overnight), and 1:2 (1 h) mixtures of propylene oxide and Epon 812 (Electron Microscopy Sciences) embedding medium, respectively. The pellets were transferred to 100% epoxy resin and incubated overnight. Infiltrated samples were embedded in fresh pure epoxy resin and polymerized for 24–48 h at 70°C. Using a Leica EM UC6 Ultramicrotome (Leica Microsystems GmbH), polymerized blocks were cut into thin slices and mounted on 200- mesh carbon-coated grids. The grids were post-stained with 2% uranyl acetate and 1% lead citrate at room temperature for 15 and 5min, respectively. Zeiss LEO912AB 120 kV TEM (Carl Zeiss) and FEI Tecnai G2 Spirit Twin 120 kV TEM (FEI Company) were used for transmission electron microscopy analysis [24].

### Relative Gene Expression at the Transcriptional Level in Host Cells in Response to HuNoV Replication

Quantitative RT-PCR was also used to measure the gene expression levels in response to HuNoV replication in the MDCK cells. RNA was extracted using 200 μl of RNAiso Plus (TaKaRa) added to the cells at the infection endpoint, according to the manufacturer's manual. For cDNA synthesis, 10 μl of RNA and 1 μl of 6-mer random primer (TaKaRa) were mixed and reacted at 65°C for 10 min. Then, the cDNA synthesis kit (4 μl of 5 × MMLV RTase reaction buffer + 2 μl of 5 × DTT + 2 μl of dNTP + 0.5 μl of RNase inhibitor + 1 μl of MMLV, TaKaRa) was added, and left to react at 37°C for 1 h and then at 95°C for 5 min. The cDNA synthesis was performed using the PCR Thermal Cycler Dice Gradient (TaKaRa). The levels of replication-related genes in the prepared cDNA were quantified with Thermal Cycler Dice^®^ Real-Time System III (TaKaRa). The primer sequences are given in Table S1.

### Statistical Analysis

All experiments were performed in triplicate. Statistical data analysis was performed using IBM SPSS v.23 software (IBM Corp., USA). Significant differences between groups were determined by one-way analysis of variance followed by Duncan’s post-hoc test. Comparisons between two groups were performed with Student’s *t*-test. A value of *p* < 0.05 was considered statistically significant.

## Results

### Optimal Cell Culture Conditions for HuNoV Replication

**Fig. 1:**
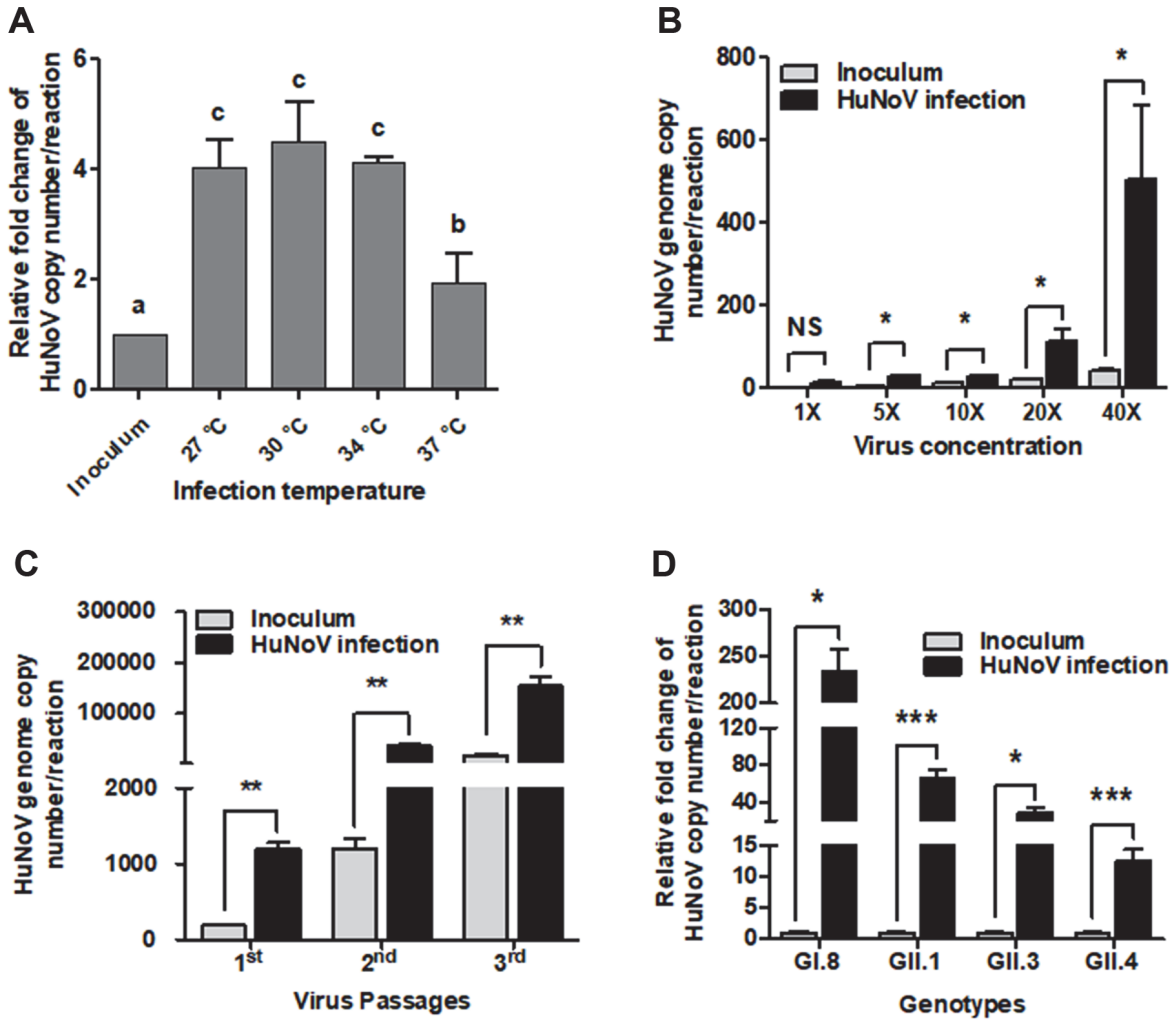
Replication of human norovirus (HuNoV) in cell lines. (**A**) HuNoV (GII.4 genotype) replication in MDCK cells at different temperatures for 72 h. (**B**) Evaluation of HuNoV replication efficiency in MDCK cells by the variation of HuNoV concentration (1X, 5.6; 5X, 2.8 × 10^1^; 10X, 5.6 × 10^1^; 20X 1.14 × 10^2^; and 40X, 2.28 × 10^2^ HuNoV genome copies). (**C**) Evaluation of the passaging of HuNoV in MDCK cells on three consecutive passages. (**D**) Replication of different HuNoV genotypes (2.28 × 10^2^ HuNoV genome copies) in MDCK cells at 30°C for 72 h. Inoculum, virus inoculated MDCK cells after 1 h incubation and washing with PBS. Each bar represents the average of triplicate samples. Letters or asterisks indicate statistical differences among or between groups determined using ANOVA followed by Duncan’s (*p* < 0.05) or Student’s *t*-test (*p < 0.05, **p < 0.01 and ***p < 00.1), respectively.

To investigate the optimal temperature for viral replication in MDCK cells, HuNoV (GII.4 genotype)-infected MDCK cells (hereafter denoted as ‘infected cells’) were seeded at a density of 6.25 × 10^4^ cells/ml and incubated in a 48-well plate at 27°C, 30°C, 34°C, and 37°C for 72 h (Fig. 1A). HuNoV replication was determined by measuring the RNA quantity of HuNoV VP1 gene in the infected cells using primers designed to amplify the VP1 [23]. The relative HuNoV copy numbers were 4.0-, 4.5-, 4.1-, and 1.9-fold higher than that of the initial inoculum at incubation temperatures of 27°C, 30°C, 34°C, and 37°C, respectively (Fig. 1A). The highest virus replication was observed at 30°C, and the lowest replication was at 37°C. Previous studies have typically used 37°C as the incubation temperature for in vitro virus culture and were therefore possibly not seeing replication [4]. To identify the optimal inoculum concentration for virus replication, the increase in the HuNoV genome copy number was measured at various concentrations of the virus inoculum (5.6 − 2.28 × 10^2^ HuNoV genome copies) in a 48-well plate. The HuNoV genome copy number was increased with increasing virus concentration, except for at 5.6 HuNoV genome copies (Fig. 1B). The highest fold-increase in the genome copy number was a 12.6-fold increase of inoculum concentration of 2.28 × 10^2^ HuNoV genome copies, followed by 5.7-, 5.6-, 3.0-fold increase of inoculum concentration of 1.14 × 10^2^ ), 2.8×10^1^ and 5.6 × 10^1^ HuNoV genome copies, respectively (Fig. 1B). To confirm the scalability of virus production and passaging of HuNoV, the infected cells were incubated in different culture dishes, and the HuNoV copy number was measured. The infected cells were used as inoculum source for a series of three passages, meaning that the infected cells from the previous passage were used as an inoculum source for the following passage. Fig. 1C shows that the HuNoV genome copy number was increased by 6.3-, 30.6-, and 8.4-fold relative to that of the corresponding initial inoculum (1.91 × 10^2^, 1.21 × 10^3^, and 1.85 × 10^4^ HuNoV genome copies) of the three consecutive passages, respectively. These results indicated that the passaging of HuNoV and scaling up of the virus production is possible in our culture system. Additionally, to identify the adaptability of the culture system, the replication of different HuNoV genotypes including GI.8, GII.1, GII.3, and GII.4 (2.28 × 10^2^ HuNoV genome copies) were observed at 30°C for 72 h (Fig. 1D). Increases of 173-, 67-, 29-, and 13-fold in the virus copy number of GI.8, GII.1, GII.3, and GII.4, respectively, were observed relative to the initial inoculum level, demonstrating the high adaptability of the culture system for different genotypes. Although the replication of GII.4 was comparatively lower than that of the other genotypes, this genotype was selected for further experiments because it is the most common cause of global outbreaks [25].

### Expression of Genes Related to HuNoV Replication at Low Temperature

**Fig. 2:**
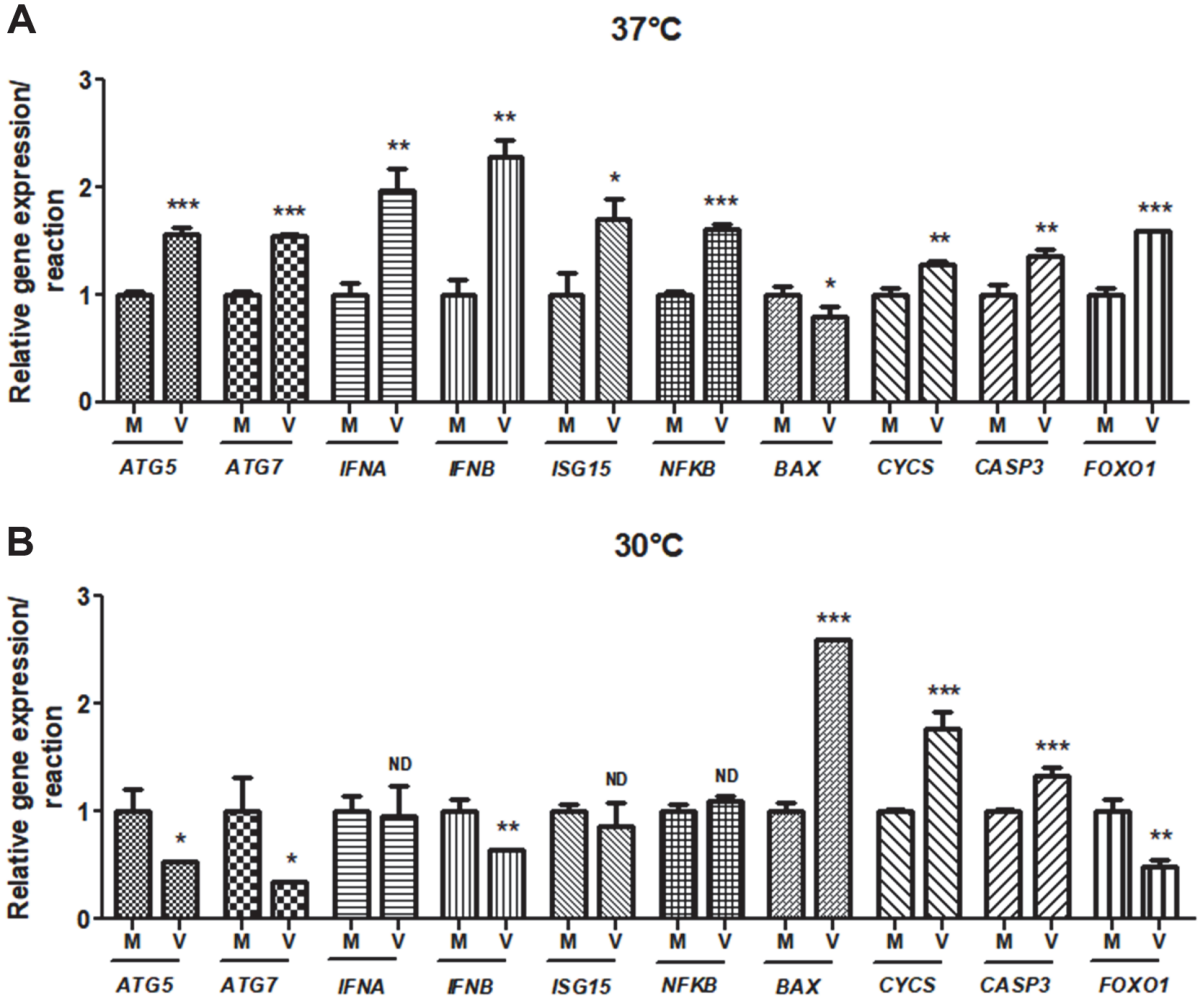
Expression of genes in human norovirus (HuNoV)-infected MDCK cells at (A) 37°C and (B) 30°C Relative expression of *ATG5*, *ATG7*, *IFNA*, *IFNB*, *ISG15*, *NFKB*, *BAX*, *CYCS*, *CASP3*, and *FOXO1* in the cells. MDCK cells were cultured for 24 h, and infected with 1.92 ×10^2^ HuNoV genome copies at 37°C and 30°C for 24 h. The gene expression was assessed by qRT-PCR and expressed relative to the expression in mock infected control. Asterisks above bars indicate the significant difference (*p < 0.05, **p < 0.01, ***p < 0.001) when compared to the corresponding gene expression in mock infected cells determined using *t*-test.

To determine the mechanism for HuNoV replication at low temperature, the expression of different genes involved in viral replication at 30°C and 37°C were analyzed and compared to the level of respective genes in a mock-infected control that was prepared with thermally inactivated (80°C for 30 min) HuNoV obtained from fecal sample. The expression level of autophagy-related gene *ATG5* was decreased by 1.9-fold at 30°C; however, the level was increased by 1.6-fold at 37°C in infected cells compared to the mock (Figs. 2A and 2B). A similar trend was observed for *ATG7* (2.84-fold decreased at 30°C and 1.5-fold increased at 37°C) (Figs. 2A and 2B). Our results indicated that at 37°C, *ATG5* and *ATG7* were induced and activated autophagy, resulting in virus degradation; thus, virus replication was reduced [26]. In contrast, at 30°C, *ATG5* and *ATG7* expression levels were reduced, thereby preventing the decrease in autophagy in the antiviral response and thus supporting virus replication at low temperature [27]. Moreover, the expression of interferon (*IFN*)*A*, *IFNB*, *IFN*-stimulated gene (*ISG)15*, and nuclear factor-kappa B (NF-κB) was upregulated by 2.0-, 2.3-, 1.7-, and 1.6- fold, respectively, at 37°C compared to that in their respective mock (Fig. 2A). Upregulation of the immune-related genes at 37°C could contribute to controlling the virus replication. However, either IFNB was downregulated or IFNA, ISG15 and NF-κB were not significantly different at 30°C compared to mock, thus allowing virus replication in infected cells at low temperature. The expression levels of genes associated with apoptosis, apoptosis regulator BAX (*BAX*), was decreased at 37°C and exceptionally increased at 30°C compared to mock. Even though other apoptosis-related genes, including cytochrome c (*CYCS*) and caspase *(CASP-3)* were significantly increased at both temperatures compared to mock, the pattern of the expression level was higher at 30°C compared to that at 37°C (Figs. 2A and 2B). Since *FOXO1* regulates several immune and autophagy related genes [28, 29] and inhibits virus replication [30], the expression of *FOXO1* was analyzed in the infected cells. The result showed that the expression level of *FOXO1* was decreased by 2.1-fold at 30°C in contrast, and was increased by 1.6-fold at 37°C in infected cells compared to mock (Figs. 2A and 2B).

Overall, these results suggest that at 37°C, autophagy- and immune-related genes were upregulated in infected cells to promote the antiviral response to HuNoV infection; however, these genes were either inhibited or unchanged at 30°C, thereby permitting viral replication.

### Inhibition of *FOXO1* Expression for HuNoV Replication

**Fig. 3:**
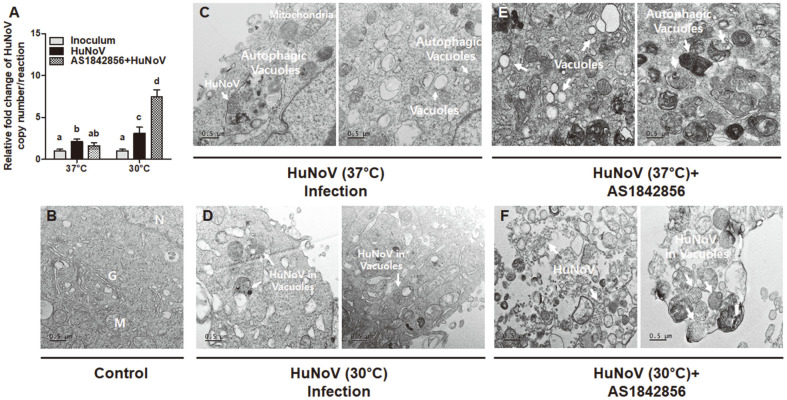
Effect of *FOXO1* inhibitor (AS1842856) in human norovirus (HuNoV) infection. (**A**) Change in relative fold of HuNoV copy number affected by *FOXO1* inhibitor (AS1842856, 20 μM) at 37°C and 30°C in MDCK cells. Statistical difference was assessed using one-way ANOVA followed by Duncan’s test (*p* < 0.05). (**B**-**F**) Transmission electron micrographs of uninfected control and HuNoV1.92×10^2^ HuNoV genome copies) infected MDCK cells treated with *FOXO1* inhibitor (AS1842856, 20 μM) at 37°C and 30°C for 72 h. (**B**) Control group showing the presence of normal organelles such as Golgi bodies (G), mitochondria (M), and nuclei (N). (**C**) Experimental group HuNoV-infected cells at 37°C, with the presence of HuNoV surrounded by autophagic vacuoles. (**D**) Experimental group HuNoV-infected cells at 30°C, with accumulation of HuNoV in vacuoles and cytoplasm. (**E**) Experimental group *FOXO1* inhibitor-treated and HuNoV-infected cells at 37°C. (**F**) Experimental group *FOXO1* inhibitor-treated and HuNoV-infected cells at 30°C. Inoculum, virus inoculated MDCK cells after 1 h incubation and washing with PBS.

Next, the effect of *FOXO1* inhibition on HuNoV replication was investigated by treating the infected cells with a *FOXO1* inhibitor (AS1842856) [26] at 30°C and 37°C. The HuNoV copy number was markedly increased by 7.5-fold following treatment with the inhibitor relative to that of the initial inoculum at 30°C; however, replication was not significantly different at 37°C (Fig. 3A). The results indicate that *FOXO1* inhibition only induced HuNoV replication at 30°C.

To investigate the effect of *FOXO1* inhibition on the cellular morphology of infected cells, ultrastructural analysis of *FOXO1* inhibitor (AS1842856)-treated HuNoV-infected and uninfected cells was performed by transmission electron microscopy. Uninfected cells exhibited well-preserved cellular morphology with evenly distributed cell constituents, such as an intact nucleus (N), Golgi apparatus (G), and mitochondria (M) (Fig. 3B). The infected cells at 37°C displayed an increase in distinct membrane-bound autophagic vacuoles containing intracellular digested organelles [31]. Similar types of autophagic vacuole were observed in poliovirus and rhinovirus [32]. Such autophagic vacuoles were visualized in both *FOXO1* inhibitor treated- and untreated- HuNoV infected cells at 37°C (Figs. 3C and 3E). However, the destruction of cellular compartments and cytoplasmic clearing were distinctly observed in *FOXO1* inhibitor treated-HuNoV infected cells at 30°C (Figs. 3D and 3F). Interestingly, in *FOXO1* inhibitor treated- HuNoV infected cells at 30°C, HuNoV, with a size of approximately 40 nm, was more abundantly and clearly detected and was located either individually or in groups (Fig. 3F). Overall, the results suggested that the inhibition of *FOXO1* may induce HuNoV replication by reducing the formation of autophagic vacuoles in the infected cells at low temperature. In addition, HuNoV infection affected cellular destruction, leading to distinctive ultrastructural features of apoptosis, whereas cell damage appeared to be more severe in cells infected at 30°C than in those infected at 37°C (Figs 3D and 3E).

## Discussion

In this study, a temperature-optimized cell culture model was developed and used to successfully replicate HuNoV using MDCK cells. A mechanism for replication in the model was also proposed. The successful replication of HuNoV in B cells and HIE in vitro were reported previously, with the cells requiring the additional cofactor HBGA and bile salt, respectively. However, Karst *et al*. reported that HBGA expression alone is not sufficient to enable virus replication in cell culture [2]; other several factors may affect virus replication. Moreover, genotypes GII.1 and GII.4 cannot bind to HBGA, but other numerous non-HBGA ligands have the potential to bind the genotypes [33, 34]. The role of cell receptors in HuNoV replication remains controversial. For example, the presence of receptors such as sialoglycan does not support replication of HuNoV in the cells [19, 35]. Therefore, several factors, such as virus genotype and cell culture condition, may be responsible for virus replication in vitro.

All HuNoV genotypes did not replicate equally in the same culture model. In our model, the highest replication was observed for GI.8 and the lowest for GII.4. However, the model successfully replicated all tested genotypes, GI.8, GII.1, GII.3, and GII.4, indicating broad adaptability of the model (Fig. 1D). Similarly, Ettayebi *et al*. demonstrated successful replication of different genotypes in the HIE model in the presence of bile salt. However, a jejunal HIE model could only replicate GII genotypes [35]. The increase in virus copy number seems to be lower in our MDCK cell model than that in HIEs [8, 35], which could be due to lower concentration of virus inoculum used for the study. Increasing virus concentration (2.28 × 10^2^ HuNoV genome copies) in inoculum increased by 12.6-fold the virus copy number in our model, indicating that the HuNoV replication also depends on viral concentration of inoculum. Moreover, modifying the cell culture conditions by lowering the temperature significantly increased HuNoV replication (Fig. 1A). Similarly, rhinovirus, a common cold virus, showed higher replication at a lower temperature (33°C) than at 37°C because of reduced antiviral immune responses [20]. Chikungunya virus also showed higher replication at 22°C than at 30°C because INF expression was reduced [19]. Thus, replication at low temperature may be explained by variations in gene expression in the cell culture affected by temperature.

All tested autophagy-related genes, including *ATG5* and *ATG7*, showed significantly increased expression levels in infected cells at 37°C, suggesting that the expression of these genes suppressed virus replication. The genes protect cells from viral infection and support the maintenance of cellular homeostasis [36](Nardacci, Amendola, Ciccosanti, Corazzari, Esposito, Vlassi, *et al*., 2014). However, the role of autophagy was inconsistent in viral replication; viruses can manipulate autophagy signaling in either a suppressive or supportive manner [37]. Consistent with our results, previous studies reported that autophagy suppressed HIV-1, Sindbis, Epstein-Barr, and herpes simplex virus replication by trapping viral components or promoting the immune response [36, 38- 41]. In contrast, autophagy supported the replication of other viruses, such as hepatitis C, dengue, varicella-zoster, and influenza A viruses, as these viruses trigger autophagosome formation to facilitate their own replication [42- 45]. At 30°C, decreased ATGs in HuNoV-infected cells indicated that inhibition of autophagy activates virus replication in the cells, possibly via inhibition of the autophagy-mediated antiviral immune system [37]. Hwang et al. also explained the role of the ATG complex in the IFN-mediated antiviral immune response against murine NoV for virus inhibition [46].

In the present study, upregulation of immune-related genes, including *IFNA*, *IFNB*, *ISG15*, and NF-κB, in infected cells at 37°C, reflected activation of their antiviral role against HuNoV, thus suppressing virus replication. A previous study also demonstrated that IFN expression suppressed the replication of different viruses, including murine NoV [47]. On the other hand, unlike immune cell and in vivo studies, MDCK cells could not demonstrate IFN-induced antiviral activity against influenza virus, which could subsequently lower antiviral defense in the cells, and promote virus replication [48, 49]. Furthermore, *ISG15* regulates the *IFN*-mediated antiviral immune response against different viruses, including murine NoV, influenza A, hepatitis C, and dengue viruses by covalently conjugating (ISGylation) to viral or cellular proteins to inhibit virus replication [50, 51]. On the other hand, either the suppression or unalteration of these immune-related genes at low culture temperature (30°C) may increase virus replication in the cells [52]. Similarly, as described previously, rhinovirus chikungunya virus was reported to replicate at lower temperatures by suppressing the activity of IFNs and/or ISGs [19, 20]. Inhibition of *ISG15* significantly increased the replication of influenza A, Sindbis, and herpes viruses [53], and inhibition of the antiviral immune response of *IFNs* and *ISGs* is critical for virus replication [54]. Moreover, NF-κB regulates the expression levels of various immune responses against the virus [55]. Consistent with the results of our study, decreased homologous NF-κB activates the viral gene expression of HIV-1 and herpes simplex virus in cells by reducing host immune and inflammatory responses [56].

BAX was exceptionally induced at 30°C in the infected cells, resulting in apoptosis possibly through activation of *CASP3* and *CYCS* at 30°C [57, 58]. Similarly, apoptosis in murine NoV-infected cells was reported to increase during virus replication via activation of *CASP3* [59]. Moreover, functional apoptosis of cells caused by HuNoV infection was confirmed by TEM. In previous studies, HuNoV particles were observed in TEM images; however, their sizes were smaller than those observed in our study [8].

Finally, *FOXO1* inhibition at low temperature induced HuNoV replication. Consistent with our result, *FOXO1* inhibition also induced HIV and Kaposi’s sarcoma-associated herpes virus replication in cells by inducing intracellular ROS levels [30, 60, 61]. However, the effect of culture temperature on *FOXO1* expression was not observed previously. Notably, *FOXO1* inhibition at low temperature may play a crucial role in virus replication because it modulates autophagy- and immunity-related genes and associated pathways [62].

In conclusion, this study demonstrated that HuNoV infection at 30°C can support virus replication in MDCK cells. The study revealed an unexpected link between temperature and the expression of *FOXO1*, which facilitated HuNoV replication in the cells. Thus, temperature-optimized MDCK cells can serve as a useful model for further studies to elucidate the pathogenic mechanisms of HuNoV infection.
